# Supplementary material for: Reconciling Mining with the Conservation of Cave Biodiversity: A Quantitative Baseline to Help Establish Conservation Priorities
Source: PLoS One. 2016 Dec 20;11(12):e0168348. doi: 10.1371/journal.pone.0168348 (PMC5173368; doi:10.1371/journal.pone.0168348)
Supplement: S1 Dataset — (ZIP) [file pone.0168348.s002.zip › Taxa/Serra Norte/SN_2013/N4E/Lista N4E-91.pdf]

CAVIDADE N4E-0091

Classe	Ordem	Fam/Outros	Gên/Outros	Espécie	S-U	U-S	Zon.
Arachnida	Acari	(Oribatida)		sp.1		X	E
Arachnida	Acari	Eviphididae		sp.1		X	E
Arachnida	Acari	Labidostommatidae		sp.1		X	E
Arachnida	Acari	veigaiidae		sp.1		X	E
Arachnida	Amblypygi	Phrynidae	*Heterophrynus*	*longicornis*		1	E
Arachnida	Araneae	Araneidae		jovem	X	X	E
Arachnida	Araneae	Corinnidae		jovem	1		E
Arachnida	Araneae	Ctenidae		jovem		1	E
Arachnida	Araneae	Drymusidae		jovem		X	E
Arachnida	Araneae	Oonopidae		jovem	X		E
Arachnida	Araneae	Paratropididae		jovem		1	E
Arachnida	Araneae	Pholcidae	*Mesabolivar*	sp.1	X	X	E
Arachnida	Araneae	Salticidae		sp.2	X		E
Arachnida	Araneae	Salticidae		sp.7		X	E
Arachnida	Araneae	Scytodidae	*Scytodes*	jovem		1	E
Arachnida	Araneae	Segestriidae		jovem	X		E
Arachnida	Araneae	Tetragnathidae		jovem		X	E
Arachnida	Araneae	Theridiosomatidae	*Plato*	sp.1	X	X	E
Arachnida	Opiliones	Cosmetidae	*Roquettea*	*singularis*	4	2	E
Arachnida	Opiliones	Sclerosomatidae	*Prionostema*	sp.1	1		E
Arachnida	Opiliones	Stygnidae	*Protimesius*	*laevis*	3		E
Arachnida	Schizomida	Hubbardiidae		jovem		X	E
Chilopoda				jovem		1	E
Diplopoda	Polydesmida	Chelodesmidae		sp.3		1	E
Diplopoda	Polydesmida	Fuhrmannodesmidae		sp.1		X	E
Diplopoda	Polydesmida	Paradoxosomatidae		sp.1		2	E
Diplopoda	Polydesmida	Pyrgodesmidae		sp.1		X	E
Entognatha	Collembola	Entomobryidae		sp.1	X		E
Entognatha	Collembola	Paronellidae		sp.1	X	X	E
Entognatha	Collembola	Sminthuroidea		sp.2		X	E
Entognatha	Diplura	Campodeidae		sp.1	X		E
Insecta	Archaeognatha	Meinertellidae		sp.1		X	E
Insecta	Blattodea			jovem	1		E
Insecta	Coleoptera	Staphylinidae	Pselaphinae	sp.34	X		E
Insecta	Coleoptera	Staphylinidae		sp.1		X	E
Insecta	Coleoptera	Staphylinidae		sp.38		X	E
Insecta	Coleoptera	Staphylinidae		sp.47		X	E
Insecta	Diptera	Cecidomyiidae	Cecidomyiinae	sp.	X		E
Insecta	Diptera	Chironomidae		sp.	X		E
Insecta	Diptera	Culicidae	*Culex*	sp.	X		E
Insecta	Diptera	Dolichopodidae		sp.	X		E
Insecta	Diptera	Phoridae	Metopininae	sp.	X		E
Insecta	Diptera	Tipulidae	Tipulinae	sp.	X	X	E
Insecta	Heteroptera	Reduviidae	Emesinae	sp.8	1		E
Insecta	Heteroptera	Reduviidae	Reduviinae	sp.		1	E
Insecta	Homoptera	Cixiidae		sp.1		X	E
Insecta	Homoptera	Cixiidae		sp.3		X	E
Insecta	Hymenoptera	Formicidae	*Brachymyrmex*	sp.1		X	E
Insecta	Hymenoptera	Formicidae	*Camponotus*	sp.2	X		E

Insecta	Hymenoptera	Formicidae	*Crematogaster*	sp.1	X		E
Insecta	Hymenoptera	Formicidae	*Eurhopalothrix*	sp.1		X	E
Insecta	Hymenoptera	Formicidae	*Pachycondyla*	sp.2	X	X	E
Insecta	Hymenoptera	Formicidae	*Paratrechina*	sp.1	X	X	E
Insecta	Hymenoptera	Formicidae	*Solenopsis*	sp.1		X	E
Insecta	Isoptera	Termitidae	*Nasutitermes*	sp.	X	X	E
Insecta	Lepidoptera	Noctuoidea		sp.1		2	E
Insecta	Lepidoptera	Noctuoidea		sp.5		1	E
Insecta	Orthoptera	Phalangopsidae	*Paraclodes*	jovem	6	3	E
Insecta	Psocoptera	Epipsocidae		jovem	X		E
Insecta	Psocoptera	Psyllipsocidae		jovem		X	E
Gastropoda	Pulmonata	Systrophiidae	*Happia*	sp.		X	E
Malacostraca	Isopoda	Armadillidae		sp.1		X	E
Malacostraca	Isopoda	Philosciidae		sp.1		X	E
Symphyla		Scutigerellidae		sp.		X	E
Amphibia	Anura	Strabomantidae	*Pristimantis*	cf. *fenestratus*	1	1	E
Mammalia	Chiroptera	Emballonuridae	*Peropteryx*	*kappleri*		1	E
